# Attachment Styles, Various Maternal Representations and a Bond to a Baby

**DOI:** 10.3390/ijerph17103363

**Published:** 2020-05-12

**Authors:** Anna Zdolska-Wawrzkiewicz, Magdalena Chrzan-Dętkoś, Daria Pizuńska, Mariola Bidzan

**Affiliations:** Institute of Psychology, Faculty of Social Science, University of Gdansk, Bazynskiego 4, 80-952 Gdansk, Poland; psymcd@ug.edu.pl (M.C.-D.); daria.pizunska@ug.edu.pl (D.P.); mariola.bidzan@ug.edu.pl (M.B.)

**Keywords:** attachment, relationship with a child, self-representation, relationship with a mother, transmission of attachment

## Abstract

(1) *Background*: The aim of this study was to assess the relationship between: (a) new mothers’ styles of attachment to their own mothers with their representation of self as a mother as well as with their representation of one’s mother as a mother, (b) new mothers’ representation of self as a mother with their representation of one’s own mother as a mother, and (c) their bonds with their children and their styles of attachment to their own mothers. (2) *Methods*: A total of 86 mothers were interviewed approximately six months postpartum. The Adjective Checklist, a modified version of the Experiences in Close Relationships, and the Postpartum Bonding Questionnaire were used in the study. (3) *Results*: Analysis revealed a statistically significant relationship between the new mothers’ styles of attachment to their own mothers and both their representation of self as a mother and their representation of one’s mother as a mother. The relationship between representation of self as a mother and representation of one’s mother as a mother was also statistically significant. No statistically significant relationship was observed between the style of attachment to one’s mother and the bond with one’s child six months postpartum. (4) *Conclusions*: A deeper understanding of the relationship between these variables may improve the help system directed at young mothers.

## 1. Introduction

The bond between mother and child has fascinated people for centuries. Researchers are trying to explore and understand the processes that take place during the first months of an infant’s life [1,2]. It is now known that the foundations of the bond between mother and child are formed during (or even before) pregnancy. Processes taking place within a woman change her psychology, are a basis for how she later perceives herself as a mother, and influence her bond with the child. Pregnancy, on a neurobiological level, involves radical hormone surges and biological adaptations. Pregnancy renders substantial changes in brain structure, for example, reductions in gray matter (GM) volume in regions subserving social cognition [3]. Furthermore, the GM volume changes of pregnancy predict measures of postpartum maternal attachment, which suggests an adaptive process serving the transition into motherhood. Research shows that the psychology of women during pregnancy may be associated with specific biological markers that may affect maternal behaviors postpartum as well as child development. For example, oxytocin levels in pregnancy, which in turn predict maternal behaviors postpartum [4], are associated with depression and is increasingly recognized as a factor that may influence offspring development [5]. Elevated maternal IL-6 or CRP (C-reactive protein) levels (markers of inflammation, often associated with antennal depression and anxiety) during pregnancy have been linked with adverse pregnancy and birth outcomes [6] and with new-born functional and structural brain alterations (e.g., [7,8]). Higher maternal cortisol in late pregnancy has been associated with lower infant cognitive scores at three months [9], 17 months [10], and seven years [11].

These studies show the significant and long term consequences of maternal mental state and well-being during pregnancy. It also shows the necessity of finding the protective factors as well as ways of supporting women, and indirectly, also the baby. In our study, we focused on attachment style and relationships with the mother as important factors facilitating the transitions to motherhood. According to theorists and practitioners associated with psychoanalytic approaches, the relationship with own mother-identification with the ‘maternal object’—internal representation of maternal figure [12]—is vital for women in the process of becoming accustomed to their new status as a mother [13]. Representations are a set of tendencies to behave in particular ways in intimate relationships, based on ideas, fantasies, and schemes of past experiences in daily interactions [14]

When preparing to welcome a child, in some sense, the woman is both a mother and a child—having access to both the child’s needs and the memories of her own relationship with her mother during her childhood [14]. This helps her to understand the needs of the infant and to provide it with care. The relationship between a woman and her own mother is extremely important for the formation of this capacity for mothering [15,16,17,18,19]. What the mother thinks about herself (conscious and unconscious) as a mother is formed from the representation of self as a woman and one’s mother as a mother [20]. How a woman remembers her mother is very important, as is how she experiences the care she received from her [2]. According to psychoanalytic author Helen Deutsch [21], the wellbeing of a woman during pregnancy and childbirth is associated with her inner representation of the representation of a mother. If this representation is devalued or hated, it prevents her from maintaining a positive representation of herself as a woman. Both her current relationship with her mother (e.g., help with child rearing [22]) and her memories of her mother from childhood are important in the time of the transition to motherhood [13]. John Bowlby defined the inner representations as “internal working models”: early mental representation of world of relations containing anticipated caregivers responses [1]. They play an important role in this process of transition to motherhood. One’s primary relationship with one’s main caregiver may become a template for one’s worldview and relationships with others, and its echoes can be heard in the close relationships one has in adulthood as well as relations with one’s own child [23,24,25,26].

Research shows that mothers who perceive themselves as “experiencing parental care” in childhood (i.e., those who could be described as being characterized by a secure attachment style) are more sensitive to signals communicated by their child [27,28,29]. Moreover, they may be more frequently bonded with their children in a secure way [30,31,32] and they respect boundaries (i.e., the child’s autonomy [27,30]). A balance between autonomy and intimacy is important in the intergenerational transfer of secure attachment style [22,33].

Thus, the internal working model that reflects high level of self-worth and self-care with the possibility of getting support from a supportive and caring representation of a parent may lower the stress associated with motherhood on both psychological and physiological levels [4,34], facilitate making use of social support, and make pregnancy motherhood less problematic [22]. On the other hand, new mothers with ambivalent, disorganized, or avoidant attachment style and absent, conflicted, rejecting care giver may transfer their insecure style of attachment to their children [34]. It can be said that women who have very negative representations of their caregivers find it more difficult to take care of their child and form a secure bond because they feel lonely and cannot use the support and consolation associated with the possession of a “internal working model” [21]. However, it can change over the lifespan and relationship, for example, Deutsch wrote in her memoirs, that her ability to become a mother was directly related to her relationship to the mother of her best friend [35]. Furthermore, other researchers have emphasized the importance of other relationships that are important to women [36].

This study is part of a research project concerning the influence of a woman’s relationship with her mother on the bond with her child as well as on her representation of self as a mother during pregnancy and after giving birth. The results of the first part of the study are described in an earlier publication [20]. More about the theoretical background was published in another article [37].

Four research questions were posed in this paper:

Do women who differ in their attachment style differ also in terms of representation of self as a mother six months postpartum?

Do women who differ in their attachment style differ also in terms of representation of their mothers as a mother 6 months postpartum?

Does a relationship exist between a woman’s representation of self as a mother six months postpartum and her representation of her mother as a mother?

Do women who differ in their attachment style differ also in terms of the bond with their child six months postpartum?

## 2. Methods

This study is the second stage of a larger project. The aim of the first study was to acquire knowledge about the connections between a mother attachment style in the case of pregnant women and their self-representation as a mother, the representation of their mother as a mother, and the bond with an unborn child [20] In this, the second stage included a total of 86 women aged between 23 and 41 (M = 30.55; SD = 3.775) who were interviewed about six months postpartum (the time of this contact was calculated based on the expected date of delivery). The researchers contacted the women who took part in the first stage of the study to propose continuation of cooperation. The contact was via email addresses collected during the first step. Women were asked to take part in the second step and were given a set of questionnaires.

The research was approved by the Research Ethics Committee at the Psychology Department of the University of Gdansk, Poland (No. 23/2014).

Most of the women were married (75.6%, *n* = 65), had higher education (83.7%, *n* = 72), and lived in a big city (54.7%, *n* = 47), 15.1% lived in the rural areas (*n* = 13), 7% in a small city (*n* = 6), and 23.3% lived in a city of more than 500,000 inhabitants (*n* = 20). For most women, this was their first child 86.0% (*n* = 74) and 14% of them were having their second child (*n* = 12). The majority of women gave birth on time (89.5%, *n* = 77) and gave birth to one child only (94.2%, *n* = 81). Most women gave vaginal birth (57%, *n* = 32 spontaneously and *n* = 17 by induction).

Comparison was made between those women who only completed the first data entry and those who completed both datasets. In the majority of the tested variables, there were no significant differences. The compared groups of women differed in number of children [t = −3.553; *p* < 0.001], ‘critical parent’ in self-representation as a mother [t = 2.059; *p* = 0.041], and also in the representation of one’s mother as a mother: ‘need for achievement’ [t = 2.164; *p* = 0.032], ‘need for domination’ [t = 2.254; *p* = 0.025], ‘need for understanding oneself and others’ [t = 2.073; *p* = 0.039] as well as ‘personal adaptation’ [t = 2.171; *p* = 0.031].

In this study, the following questionnaires were used:

1. The Polish adaptation [38] of the Experiences in Close Relationships (ECR) questionnaire [28]. The ECR questionnaire measures two dimensions of attachment to a partner in a romantic relationship: Avoidance and Anxiety. Based on the scores on these two dimensions, participants are classified into four attachment styles: Secure, Preoccupied, Dismissing, and Fearful. Due to the necessity of excluding some questions in the Polish adaptation (which was adjusted for retrospective measurement of a mother’s attachment style), participants were categorized in terms of two styles/dimensions: Fearful-ambivalent and Avoidant. The reliability of the scales was calculated using Cronbach’s alpha (*n* = 302, Polish sample), which equaled 0.89 and 0.68 for the scales measuring Avoidant and Fearful-ambivalent styles of attachment toward one’s mother, respectively. The validity equaled 0.45 and 0.20 for scales measuring Avoidant and Fearful-ambivalent styles of attachment towards one’s mother. The Polish adaptation consists of 19 questions. Participants answer on a 7-point Likert scale, where 1 indicates ‘I strongly agree’ and 7 indicates ‘I strongly disagree’ [38]. Marchwicki obtained the consent of the authors of the original ECR version for the adaptation of the tool. The basis for agreement was a common theoretical background.

For the purposes of this study, the participants were divided into four groups based on their scores as follows:

High Fearful-ambivalent and High Avoidant—Group 1
Low Fearful-ambivalent and Low Avoidant—Group 2
High Fearful-ambivalent and Low Avoidant—Group 3
Low Fearful-ambivalent and High Avoidant—Group 4

2. A Polish adaptation of the Adjective Checklist (ACL) by Gough and Heilbrun [39]. Women filled in this questionnaire twice. First, from the list of three hundred adjectives, they chose those that they believed best described themselves as mothers: ‘Me as a Mother’. Second, they had to choose those that they believed best described their mothers as mothers: ‘My Mother as a Mother’. The reliability of the questionnaire in the Polish sample was measured using Cronbach’s alpha, which ranged between 0.43 and 0.94. We chose ACL due to its complexity and the possibility of a multi-dimensioned approach to personality. Correlations of ACL scales with other personality measures (Eysenck Personality Questionnaire-Revised, Eysenck Impulsiveness Questionnaire, State-Trait Anxiety Inventory, Minnesota Multiphasic Personality Inventory-2, KPD-Depresion Questionnaire) as well as a comparison of the results obtained by people from different clinical and professional groups and differing demographic factors confirmed the diagnostic significance of most scales [39]. The descriptions of scales are available in the manual [40].

3. The Postpartum Bonding Questionnaire [41] is used in the perinatal period to measure the relation between mother and infant. This tool consists of four subscales:

Impaired Bonding (“My baby cries to much”);
Rejection and Pathological Anger (“I feel distance from my baby”);
Infant-focused Anxiety (I feel confident while caring for my baby”); and
Incipient Abuse (“I have done harmful thinks to my baby”).

These scales describe the bond between a mother and a child [42]. The reliability of the questionnaire was measured using Cronbach’s alpha, which was 0.886.

4. A self-developed socio-demographic questionnaire was used to collect socio-demographic data.

## 3. Results

All statistical analyses were performed using SPSS 25.0 (IBM, Chicago, IL, USA) software packages. In order to answer the first, second, and last questions, the authors conducted the Kruskal–Wallis H test. Four factor levels of the attachment style were obtained using the medians of anxious–ambivalent and avoidance scales in the Experiences in Close Relationships questionnaire. In the third research question, the Pearson’s correlation coefficient was used.

In order to verify the first research question regarding the potential differences in the representation of oneself as a mother in women with different attachment styles to their own mother, a statistical analysis using the Kruskal–Wallis H test was performed. The results are shown in Table 1.

It revealed significant differences in women who differed in their attachment styles to their mothers in four of the 36 dimensions of -representation of oneself as a mother. There were significant differences in ‘need for autonomy’ [*χ²* = 12.56; *p* = 0.006], and ‘need for demeaning oneself’ [*χ²* = 11.87; *p* = 0.008], where a pairwise comparison revealed a difference between women characterized by high levels of anxiety and low levels of avoidance and women characterized by low levels of anxiety and avoidance. Despite finding significant differences in the ‘self-control’ dimension [*χ²* = 8.30; *p* = 0.040], the pairwise comparison test did not reveal differences between the groups of women. Women characterized by high levels of anxiety and low levels of avoidance were different to women characterized by low levels of avoidance and anxiety, and also to women characterized by low levels of anxiety and high levels of avoidance in the ‘spontaneous child’ dimension [*χ²* = 12.61; *p* = 0.006].

The second research question, regarding the differences in the representation of one’s mother as a mother six months postpartum depending on their style of attachment to their mothers, was investigated using the Kruskal–Wallis H test, with the results in Table 2.

It was shown that women differed in terms of their representation of their mothers as mothers, depending on their attachment style to their own mothers, in 14 out of 36 dimensions: ‘positive adjectives’ [*χ²* = 9.64; *p* = 0.022], ‘negative adjectives’ [*χ²* = 13.89; *p* = 0.003], ‘typicality’ [*χ²* = 14.07; *p* = 0.003], ‘need for domination’ [*χ²* = 9.75; *p* = 0.021], ‘need for caring’ [*χ²* = 14.24; *p* = 0.003], ‘need for affiliation’ [*χ²* = 9.16; *p* = 0.027], ‘need for heterosexual contacts’ [*χ²* = 16.93; *p* = 0.001], ‘self-confidence’ [*χ²* = 12.55; *p* = 0.006], ‘perfect me’ [*χ²* = 9.93; *p* = 0.019 ], ‘leadership’ [*χ²* = 13.70; *p* = 0.003], ‘femininity’ [*χ²* = 9.90; *p* = 0.019], ‘caring parent’ [*χ²* = 13.58; *p* = 0.004], ‘adult’ [*χ²* = 7.89; *p* = 0.048], and on the ‘submissive child’ subscale [*χ²* = 12.61; *p* = 0.006]. Pairwise comparisons revealed that statistically significant differences in terms of all the listed dimensions (except for the ‘adult’ dimension, where the comparison did not reveal any difference between women who differed in their attachment styles) between women with high levels of anxiety and avoidance, and women with low levels of anxiety and avoidance. Additionally, with regard to the ‘negative adjectives’ and ‘typicality’ dimensions, significant differences were observed between mothers with high levels of anxiety and avoidance, and mothers with high levels of anxiety and low levels of avoidance.

In order to verify the third research question regarding the relationship between the representation of oneself as a mother and the representation of one’s mother as a mother half year after childbirth, Pearson’s correlation coefficient was used.

The statistical analysis revealed strong positive relationships between the representation of oneself as a mother and the representation of one’s mother as a mother among the participants for the entire list of adjectives [*r* = 0.799; *p* < 0.001], ‘positive adjectives’ [*r* = 0.663; *p* <0.001], ‘need for perseverance’ [*r* = 0.515; *p* < 0.001], ‘need for order’ [*r* = 0.675; *p* < 0.001], ‘need for understanding oneself and others’ [*r* = 0.607; *p* < 0.001], ‘need for caring’ [*r* = 0.515; *p* < 0.001], ‘need for affiliation’ [*r* = 0.717; *p* < 0.001], ‘need for heterosexual contacts’ [*r* = 0.530; *p* < 0.001], ‘leadership’ [*r* = 0.555; *p* < 0.001], ‘ femininity’ [*r* = 0.623; *p* <0.001], ‘high originality–high intelligence’ [*r* = 0.645; *p* < 0.001], ‘low originality–low intelligence’ [*r* = 0.691; *p* < 0.001] and ‘low originality–high intelligence’ [*r* = 0.678; *p* < 0.001]. Significant positive correlations were also observed for ‘typicality’ [*r* = 0.483; *p* < 0.001], ‘need for achievement’ [*r* = 0.379; *p* < 0.001], ‘need for change’ [*r* = 0.326; *p* = 0.002], ‘self-confidence’ [*r* = 0.315; *p* = 0.003], ‘personal adaptation’ [*r* = 0.407; *p* < 0.001], ‘masculinity’ [*r* = 0.434; *p* < 0.001], ‘caring parent’ [*r* = 0.420; *p* < 0.001], ‘adult’ [*r* = 0.343; *p* = 0.001], ‘subordinate child’ [*r* = 0.352; *p* = 0.001], and ‘high originality–low intelligence’ [*r* = 0.475; *p* < 0.001]. The analyses also indicate a weak positive relationship between the representation of oneself as a mother and the representation of one’s mother as a mother in terms of the following dimensions:

‘Readiness for therapy’ [*r* = 0.255; *p* = 0.018], ‘self-control’ [*r* = 0.251; *p* = 0.020], and ‘perfect me’ [*r* = 0.226; *p* = 0.036]. Moreover, the analyses revealed a small negative correlation with the ‘need for autonomy’ variable [*r* = −0.227; *p* = 0.036]. These results suggest that the representation of oneself as a mother and the representation of one’s mother as a mother do not correlate in the following dimensions: ‘negative adjectives’ [*r* = 0.202; *p* = 0.063], ‘need for dominance’ [*r* = 0.141; *p* = 0.194], ‘need for mental exhibitionism’ [*r* = 0.147; *p* = 0.176], ‘need for aggression’ [*r* = 0.212; *p* = 0.050], ‘need for experiencing care and support’ [*r* = −0.117; *p* = 0.283], ‘need for demeaning oneself’ [*r* = −0.001; *p* = 0.991], ‘need for subordinating oneself’ [*r* = 0.146; *p* = 0.181], ‘creative personality’ [*r* = 0.074; *p* = 0.498], ‘critical parent’ [*r* = 0.145; *p* = 0.183], and ‘spontaneous child’ [*r* = 0.195; *p* = 0.073].

The last research question important for the project (i.e., whether women characterized by different attachment styles differ in terms of their bond with their child six months postpartum) was verified using the Kruskal–Wallis H test. Statistical analyses revealed no differences between women of different attachment styles on variables included in the Table 3 below.

## 4. Discussion

### 4.1. Our Research Questions

Do women who differ in their attachment style differ also in terms of representation of self as a mother six months postpartum?

Do women who differ in their attachment style differ also in terms of representation of their mothers as a mother 6 months postpartum?

Does a relationship exist between a woman’s representation of self as a mother six months postpartum and her representation of her mother as a mother?

Do women who differ in their attachment style differ also in terms of the bond with their child six months postpartum?

The presented results suggest a strong relationship between style of attachment to one’s own mother and one’s representation of self as a mother. The results are statistically significant for four subscales. These results can be interpreted in the context of attachment theory. Women who form secure bonds may have a sense of being worthy of attention and their significant others are able to provide them with nurturance. Having such internal working models also provides a person with adequate care in adulthood, which may be especially important in moments of change such as becoming a parent. Internalized good experiences serve as support when there is no possibility of external support [1]. Unlike people with such experiences, those who form bonds imbued with anxiety may have low self-confidence and seek guidance at any cost, as in their internal world they are not worthy of nurturance [43].

This internal world is unconscious, which is why it is difficult to discern the need to obtain adequate help. It is difficult for such people to take care of themselves, seek help and accept it, because they do not have such experiences in their relationship with their mother. Our study demonstrated a relationship between the style of attachment to one’s own mother and the representation of one’s mother as a mother. The relationship or bond with one’s mother is likely to be related to the representation of a woman has of her own mother.

The bond with one’s mother contains the representation of the mother, which creates internal working models; attachment is formed depending on how the mother is perceived by the child and through her reactions to signals from the child [1].

In our study, women characterized by a secure attachment assessed their mothers with a higher number of favorable adjectives and a lower number of negative adjectives, thinking about their mothers as both nurturing and also able to take care of themselves. They also had a need for interaction with other women, something that appears to be very important, this aids women in forming a support network, which may be very helpful to young mothers. In contrast, women who formed bonds that were characterized by more anxiety or avoidance experienced their mothers as more negative, less nurturing, and as having less self-confidence. These results are very important in the context of taking care of one’s own child. Women with an insecure attachment style, especially with more avoidance in their attachment, who have a negative representation of their mother, may have difficulty getting adequate support, an important protective factor in early motherhood [44]. This support may be crucial, both avoidance and anxiety have been linked in other studies with more negative perceptions of the parent–child relationship (e.g., feeling disliked by one’s child, [45]).

Being a new mother, they lack a very important model that they could use for support. They may even build this negative thinking about their mother into their own self-representation, thus making it a negative perception of self [21]. It is worth noting that these results are similar to the results obtained in the first stage of the study (i.e., pregnant women with insecure attachment styles to their own mothers also had more negative representations of their mothers [20]).

Our study showed a significant relationship on many scales between the representation of self as a mother and the representation of one’s mother as a mother. As previously stated, the representation of self as a mother is composed of the representation of one’s mother as a mother and the representation of self as a woman [46]. This result seems to complement the remaining two results of our study, emphasizing the importance of the relationship with one’s own mother in the process of becoming a mother.

The lack of relationship between one’s attachment style to one’s mother and the bond with one’s child is surprising.

It may be that some protective factors for the bond that were not investigated in our study were at play here. The relationship with one’s partner is probably such a factor [36,47]. Even in the case of a difficult relationship with one’s own mother, a good relationship with one’s partner may protect one from depression or from transferring an insecure attachment style to one’s child [48,49,50]. However, it should not be forgotten that the echoes of the relationship with one’s own mother are also present in the relationship with one’s partner (e.g., [51]). It could be that the lack of significant results is associated with the characteristics of the questionnaire. It is intended to assess bond formation in the perinatal period, but according to some researchers [52], it mainly assesses the levels of anxiety in child care in the first weeks postpartum.

Perhaps it is also the result of not taking into account in the study the factors that protect and make it difficult to establish a relationship with the child. Perhaps a wider vision than the research framework allows would be needed.

### 4.2. Limitations

The representation of self as a mother starts to separate from the representation of one’s mother as a mother a few months after giving birth. First-hand experience of taking care of a child makes it easier for a woman to make a distinction between the representation of self as a mother and the representation of her own mother as a mother [53]. It could be that six months postpartum is still the early stages of this separation, and thus the observed relationships were not very significant. It is, however, worth noting that the representation of one’s mother as a mother has a significant influence on the overall ‘motherhood’ of a woman [53].

The next limitation of the study is the fact that mothers were contacted six months after the expected date of delivery. This is problematic because it does not take into consideration the fact that some of the children were born prematurely, and this is an important issue [54]. Moreover we did not have data connected to the hospitalization of the children or the outcomes connected with delivery (planed or emergency).

The number of participants could be a limitation of this study. It should also be borne in mind that it may not be possible to generalize our conclusions onto a wider population because most of the participants had a higher education and lived in a big city. The influence of other people on the formation of a mother’s bond with her child was not controlled for in our study. It is worth adding that the study took place in Poland, and all of the participants were Polish, which may also limit the ability to generalize the results as culture plays an important role in the formation, intergenerational transfer, and expression of attachment patterns [46,55,56,57]. It may also be a strength of this study because not many studies like this are taking place in our country.

It was demonstrated that “the assumptions of monotropy, the conception of stranger anxiety as well as the definition of attachment in mainstream attachment research are in line with the conception of psychological autonomy, adaptive for Western middle-class, but deviate from the cultural values of many non-Western and mainly rural eco-social environments.” [58].

## 5. Conclusions

Our study showed that there is a relationship in young mothers between style of attachment to their mother and their representation of self as a mother as well as their representation of their mother as a mother. It also showed a strong relationship between the representation of self as a mother and the representation of one’s mother as a mother.

The practical implications of these results are extremely important. They seem to justify the provision of young mothers with support and care, as studies have shown that providing support and care to a young mother may protect her and help her build a bond with her child. Such support seems particularly important for mothers characterized by an insecure pattern of attachment. Interaction with an attentive, supportive individual (e.g., a midwife, physician, lactation consultant, or a support group) may be particularly important for these individuals. It is worth emphasizing the role of medical personnel (a physician or nurse) in observing the relationship between symptoms reported by a patient and her mental state in order to, inter alia, refer her for psychological consultation if necessary. According to research, the opportunity to form a relationship with a physician who plays the role of a “maternal object” leads to a decrease in the anxiety experienced by the patient, for example, mothers who received comprehensive information from their physician slept on average two hours longer and experienced fewer perinatal complications [59]. Culpepper and Jack [60] reported that medical interventions aimed at supporting a pregnancy may prove ineffective if psychological factors are not taken into account. This is also confirmed by studies on attachment styles: women with an avoidant attachment style are more prone to PTSD (Posttraumatic Stress Disorder) associated with perinatal experiences, especially if they experienced many interventions while giving birth [61]. This highlights the importance of a mother’s attachment and its influence on becoming a mother.

Help provided to a young mother would influence her relationship with the child and also, indirectly, the relationships built in the future by the child. Emotional support given by, say, midwives, could reduce stress and symptoms of depression as well as strengthen self-esteem and a sense of competence in young mothers.

It is worth noting that our study indirectly confirms the conclusions made by other researchers with regard to the appropriateness of short-term therapy for young mothers [62]. Such measures could help a woman introduce some kind of detachment toward representations of relations that are important for her, and thus influence the formation of her identity as a mother. It may be important for the professionals and other people supporting young mothers to allow an opportunity for the new mother to gently explore the relationship between her own mother and how she feels about becoming a mother.

Both the literature (e.g., [63,64]) and our study show that there is still a lot to do in the realm of the mental health of mothers and children.

## Figures and Tables

**Table 1:** Significant differences in the representation of oneself as a mother among women with different styles of attachment to their mothers.

Me as a Mother	Style of Attachment to One’s Mother				
	High Anxiety High Avoidance *N* = 24	Low Anxiety Low Avoidance *N* = 28	High Anxiety Low Avoidance *N* = 17	Low Anxiety High Avoidance *N* = 17	*χ²*
Need for Autonomy	0.39	1.64	−1.24	0.89	12.56
Need for Demeaning Oneself	1.65	−0.54	2.76	0.22	11.87
Self-control	0.39	−0.68	1.24	−0.83	8.30
Spontaneous Child	1.52	4.36	0.71	4.33	12.61

**Table 2:** Significant differences in the representation of one’s mother as a mother among women with different styles of attachment to their mothers.

My Mother as a Mother	Style of Attachment to One’s Mother				
	High Anxiety High Avoidance *N* = 24	Low Anxiety Low Avoidance *N* = 28	High Anxiety Low Avoidance *N* = 17	Low Anxiety High Avoidance *N* = 17	*χ²*
Positive Adjectives	16.35	28.29	25.76	20.33	9.64
Negative Adjectives	6.70	1.57	1.94	2.17	13.89
Typicality	1.65	5.46	4.71	3.83	14.07
Need for Domination	1.61	4.50	4.24	2.28	9.75
Need for Caring	3.70	10.57	9.35	7.11	14.24
Need for Affiliation	6.74	12.11	10.00	9.33	9.16
Need for Heterosexual Contacts	2.39	6.00	4.65	3.72	16.93
Self-confidence	2.00	6.68	5.29	3.78	12.55
Perfect me	2.13	6.29	4.59	3.94	9.93
Leadership	2.39	6.54	6.12	4.06	13.70
Femininity	3.96	7.18	6.41	5.06	9.90
Caring Parent	3.74	9.36	7.88	6.56	13.58
Adult	3.35	5.68	6.53	4.17	7.89
Submissive Child	−1.52	−5.11	−4.41	−1.89	12.61

**Table 3:** Bond with child in mothers with different styles of attachment to their mothers.

Bond with the Child	Style of Attachment to One’s Mother				
	High Anxiety High Avoidance *N* = 24	Low Anxiety Low Avoidance *N* = 28	High Anxiety Low Avoidance *N* = 17	Low Anxiety High Avoidance *N* = 17	*χ²*
Impaired Bonding	16.00	13.93	13.88	15.06	5.43
Rejection and Anger	8.87	8.00	8.29	9.06	5.10
Infant-focused Anxiety	5.09	4.86	5.18	5.33	1.35
Incipient Abuse	2.09	2.00	2.24	2.17	2.92
Bond with Child	32.04	28.79	29.59	31.61	3.87
